# Chronic exposure to Cytolethal Distending Toxin (CDT) promotes a cGAS-dependent type I interferon response

**DOI:** 10.1007/s00018-021-03902-x

**Published:** 2021-07-25

**Authors:** Benoît J. Pons, Aurélie Pettes-Duler, Claire Naylies, Frédéric Taieb, Catherine Bouchenot, Saleha Hashim, Patrick Rouimi, Maxime Deslande, Yannick Lippi, Gladys Mirey, Julien Vignard

**Affiliations:** 1 Toxalim (Research Centre in Food Toxicology), Université de Toulouse, INRAE, ENVT, INP-Purpan, UPS, Toulouse, France; 2 Institut de Recherche en Santé Digestive, Université de Toulouse, INSERM, INRAE, ENVT, UPS, Toulouse, France

**Keywords:** cGAS-STING axis, Bacterial genotoxin, DNA damage response, Interferon-stimulated genes, Micronucleus, Mitosis

## Abstract

The Cytolethal Distending Toxin (CDT) is a bacterial genotoxin produced by pathogenic bacteria causing major foodborne diseases worldwide. CDT activates the DNA Damage Response and modulates the host immune response, but the precise relationship between these outcomes has not been addressed so far. Here, we show that chronic exposure to CDT in HeLa cells or mouse embryonic fibroblasts promotes a strong type I interferon (IFN) response that depends on the cytoplasmic DNA sensor cyclic guanosine monophosphate (GMP)-adenosine monophosphate (AMP) synthase (cGAS) through the recognition of micronuclei. Indeed, despite active cell cycle checkpoints and in contrast to other DNA damaging agents, cells exposed to CDT reach mitosis where they accumulate massive DNA damage, resulting in chromosome fragmentation and micronucleus formation in daughter cells. These mitotic phenotypes are observed with CDT from various origins and in cancer or normal cell lines. Finally, we show that CDT exposure in immortalized normal colonic epithelial cells is associated to cGAS protein loss and low type I IFN response, implying that CDT immunomodulatory function may vary depending on tissue and cell type. Thus, our results establish a direct link between CDT-induced DNA damage, genetic instability and the cellular immune response that may be relevant in the context of natural infection associated to chronic inflammation or carcinogenesis.

**Supplementary Information:**

The online version contains supplementary material available at 10.1007/s00018-021-03902-x.

## Introduction

Cytolethal Distending Toxin (CDT) is a bacterial genotoxin produced by more than 30 phylogenetically distant Proteobacteria, including pathogenic strains responsible for major inflammatory diseases all over the world, like *Escherichia coli* (*E. col*), *Campylobacter jejuni* (*C. jej*), *Salmonella enterica*, *Shigella spp.*, *Haemophilus ducreyi* (*H. duc*), *Aggregatibacter actinomycetemcomitans* (*A. act*) or *Helicobacter spp.* [1]. In 2010, CDT-producing bacteria caused 260 million foodborne illnesses worldwide, either diarrhoeal or invasive infectious diseases, resulting in 200,000 deaths [2]. While the current model supports that CDT modulates the host immune microenvironment to promote persistent bacterial colonization, the precise role of CDT in pathogenicity is still to be elucidated.

Several lines of evidence suggest that CDT alters the host immune microenvironment in the colonized tissues during bacterial infection. First, more than 80% of *H. duc* and *A. act* strains, respectively, responsible for chancroid lesions and periodontitis, harbor the CDT operon [3]. In *C. jej*, the most common reported cause of human bacterial gastroenteritis, the prevalence of CDT genes is higher in strains isolated from diarrhoeal stool samples, reaching 89–99% [4–6]. More generally, post-infectious irritable bowel syndrome from bacterial origins are attributable to four CDT-producing bacteria: *Campylobacter spp., Salmonella enterica*, *Shigella spp.* or *E. col* [7]. Antibodies against the CDT catalytic subunit are frequently produced during inflammatory bowel disease (IBD) with diarrhea and have thus been proposed to serve as biomarkers [8]. CDT proinflammatory effects have also been depicted in rodent models with genetically altered immune responses. Only CDT-positive *Helicobacter hepaticus* (*H. hep*) were able to promote typhlocolitis in the *IL10*^*−/−*^ mice model for IBD [9]. Moreover, while an active CDT toxin slightly enhances gastritis in wild-type mice infected by *C. jej*, this effect is much more pronounced in absence of NF-κB [10].

CDT-mediated immune response activation has been mainly studied through the release of proinflammatory mediators. In mice, production of an active CDT by *H. hep* promotes the hepatic expression of TNFα, IFN-γ, Cox2, Interleukin-6 (IL-6) and the NF-κB subunits p50 and p65 4 months post-infection [11]. Interestingly, these mice develop hepatic dysplasic nodules 10 months post-infection, questioning on possible link between CDT-mediated chronic inflammatory response and tumorigenicity. Numerous in vitro studies have confirmed that CDT-intoxicated cells produce proinflammatory cytokines [12]. When exposed to *A. act* CDT, peripheral blood mononuclear cells secrete IL-1β, IL-6, IL-8 and IFN-γ [13] and macrophages secrete IL-1β, IL-6 and TNFα [14]. Induction of proinflammatory cytokines after CDT is not restricted to immune cells. Human colonic epithelial cells treated with *C. jej* CDT produce IL-8 in a NF-κB-dependent manner [15]. In the same way, intestinal or colorectal cell lines treated with *H. hep* CDT produce IL-8 and overexpress numerous NF-κB-related genes, including genes responsive to type I interferon (IFN) [16]. Understanding CDT immunomodulatory properties is thus necessary to unravel its role during bacterial infection.

CDT is composed by three subunits, CdtA, CdtC and the catalytically active CdtB, except for *Salmonella enterica* for which CdtB is associated to PltA and PltB to form the typhoid toxin [17, 18]. Whatever the toxin structure, CdtB catalytic activity is essential to mediate CDT-related cellular defects and pathogenicity [19]. CdtB belongs to the cation-dependent endonuclease–exonuclease–phosphatase (EEP) superfamily and has been structurally and functionally related to DNase I [17, 20]. Numerous studies have reported that the genotoxic activity exerted by CDT in a broad range of host cell lineages controls their outcome [21]. Remarkably, CDT-exposed cells accumulate DNA strand breaks and, therefore, activate the DNA Damage Response (DDR) through ATM-CHK2 and ATR-CHK1 signaling pathways to promote cell cycle arrest and apoptotic cell death [22–24]. Cells that survive the acute phase of intoxication enter a senescent state, characterized by a permanent cell cycle arrest, persistent DDR activation, enhanced beta-galactosidase (β-Gal) activity and cell distension [25]. Another feature of senescent cells, the senescence-associated secretory phenotype consisting in the production of cytokine [26], has also been documented after CDT-induced senescence, notably the proinflammatory mediators IL-1β, IL-6 and IL-8.

It is, therefore, tempting to speculate on a correlation between DNA damage, senescence and the proinflammatory signatures generally observed in response to CDT. Indeed, unrepaired DNA lesions can result in the formation of micronuclei (MN) after cell division, inducing a proinflammatory response once sensed as cytosolic DNA by the cyclic guanosine monophosphate (GMP)-adenosine monophosphate (AMP) synthase (cGAS) [27, 28]. Activated cGAS then synthetizes cyclic GMP-AMP, a second messenger that binds the Stimulation of Interferon Genes (STING) adaptor to activate TBK1, IRF3 and NF-κB, eliciting a type I IFN signature and promoting senescence [29].

However, in a *Salmonella enterica* infection model of immunocompetent mice, typhoid toxin production promotes an intestinal anti-inflammatory environment despite inducting DNA damage and senescence [30]. Indeed, only active CdtB harboring toxins were able to repress the inflammatory response associated with this serovar [31]. Therefore, alike typhoid toxin, the relationship between CDT-induced DNA damage and the host immune response is probably more intricate than merely activating the proinflammatory response as classically observed in vitro or with immunodeficient or cancer-prone animal models. In light of this, *H. hep* CDT can induce the expression of *IFNB1* [16], encoding type I IFN that present anti-inflammatory properties [32].

Here, we investigated the role of cGAS in CDT-induced modulation of the cellular immune response. Chronic exposure to CDT induces MN accumulation bound by cGAS, and promotes a proinflammatory response and activation of type I IFN signaling associated to a strong interferon-stimulated genes (ISGs) overexpression. Strikingly, cGAS-STING regulate ISGs but not proinflammatory cytokine expression in HeLa cancer cells and primary mouse embryonic fibroblasts (MEFs). This response depends on CdtB catalytic activity and DNA damage induction. In contrast to other genotoxic compounds, cells exposed to CDT do not primarily block at G2/M checkpoint of the cell cycle but progress through mitosis, where they suffer massive DNA damage leading to chromosome fragmentation and missegregation. This atypical scenario, observed with CDT from various origins in cancer or normal cell lines, results in MN formation within daughter cells. Finally, we present a distinct situation in immortalized normal human colonic epithelial cells (HCECs) in which the CDT-mediated diminution of cGAS protein level is associated to a low and cGAS-STING-independent type I IFN response. As MN are drivers of genetic instability and may sustain chronic cell-mediated immune response through the cGAS-STING axis, these findings suggest that CDT modulates the host immune microenvironment and promotes tumorigenic processes through MN formation.

## Materials and methods

### Cell culture and treatments

HeLa cells (HeLa-S3 cells, ATCC) and Mouse Embryonic Fibroblasts (MEFs, kindly provided by Dr. Patrick Calsou, France) were cultured in DMEM (Life Technologies), supplemented with 10% heat-inactivated fetal bovine serum (FBS, Gibco) and 1% antibiotics (penicillin/streptomycin). Human colonic epithelial cell (HCECs), generated and provided by Pr Jerry W Shay, were cultured as previously described [37] in 4:1 high-glucose DMEM/medium 199 supplemented with 2% FBS, epidermal growth factor (20 ng/ml), hydrocortisone (1 mg/ml), insulin (10 mg/ml), transferrin (2 mg/ml), sodium selenite (5 nM), and Gentamycin sulfate (50 μg/ml). Cells were maintained at 37 °C in a humidified atmosphere containing 5% CO_2_, and subcultured every 2–3 days.

*E. col* CDT (WT or the catalytic dead mutant H153A) were produced and purified as previously described [33]. ATM inhibitor KU-55933 (ATMi), ATR inhibitor VE-821 (ATRi), STING inhibitor H-151 (STINGi) and cGAS inhibitor RU.521 (cGASi) were purchased from Selleckchem. ATMi and ATRi were used at a final concentration of 10 μM, STINGi at 0.5 μM for HCECs or 1 μM for MEFs, and cGASi at 1 μM. All other chemicals and reagents were purchased from Sigma-Aldrich.

### Western blot analyses

Cells were incubated on ice for 30 min in lysis buffer (50 mM Tris–HCl pH7.5, 500 mM NaCl and 0.5% NP40) containing the HaltTM Protease and Phosphatase inhibitor cocktail (Thermo Scientific) and sonicated on a VibraCell 72,434 (Bioblock Scientific). Cell lysates were centrifugated and the supernatant containing total soluble proteins was kept. Proteins were separated by SDS-PAGE and transferred to a nitrocellulose membrane (Amersham). Membranes were incubated with the primary antibody over-night at 4 °C. γH2AX antibody was purchased from Merck/Millipore (05–636), pChk1 (133D3), pChk2 (C13C1), pH3 (D2C8), cGAS (D1D3G) and pSTAT1 (58D6) antibodies from Cell Signaling, and GAPDH (GTX100118), Cleaved Caspase-1 (GTX133447), p21 (GTX629543) and ISG15 (GTX121474) antibodies from GeneTex. The secondary anti-mouse or anti-rabbit HRP-conjugated antibodies (Jackson Immunoresearch laboratories) were incubated for 1 h at room temperature. Proteins were visualized with the enhanced chemiluminescence substrate ECL (Biorad) and imaged using the ChemiDoc XRS Biorad Imager and Image Lab Software.

### Immunofluorescence analyses and micronucleus assay

Cells were grown on glass coverslips. After at least 24 h of culture, cells were fixed with 4% paraformaldehyde, permeabilized with 0.5% Triton X-100, blocked with 3% BSA and 0.05% IGEPAL, and stained with primary antibodies for 2 h at room temperature in blocking solution (all solutions were prepared in PBS). Cells were washed three times with PBS 0.05% IGEPAL and incubated with the secondary antibodies for 1 h (Alexa Fluor 488 Goat anti-mouse (A32723) or Alexa Fluor 594 anti-rabbit (A32740), purchased from Invitrogen). DNA was stained with 4.6-diamino-2-phenyl indole (DAPI) 30 nM. Coverslips were mounted onto slides with PBS-glycerol (90%) containing 1 mg/ml paraphenylenediamine and observed at 40 × magnification with a Nikon 50i fluorescence microscope equipped with a Luca S camera. Cells were counted positive for foci formation when > 10 foci/interphase nuclei or when > 5 foci/mitotic nuclei were detected.

### Cell viability assays

For colony formation assay, cells were plated in triplicate at a density of 300 to 3000 cells per well in 6 wells plate. One day after seeding, cells were treated and grown for 10 days. Formed colonies were fixed and stained with a 0.25% methylene blue and 100% methanol solution. Colonies containing more than 50 cells were counted and the surviving rate calculated. For rapid viability testing at 24 h, cells were plated in triplicate at a density of 10,000 per well in 96-well plate. One day after seeding, cells were treated and grown for 24 h. Viability was assessed using the CellTiter-Glo® Luminescent Cell Viability Assay (Promega) according to the manufacturer’s instructions.

### Senescence assay

The senescence-associated β-galactosidase staining was performed using the Senescence β-Galactosidase Staining Kit (Cell Signaling) according to the manufacturer’s instructions. Images were captured by light microscopy.

### Cell cycle analyses by flow cytometry

For 5-ethynyl-2′-deoxyuridine (EdU) labeling at early S phase, cells were first synchronized at G1/S by double thymidine bloc. Cells were treated for 20 h with 2 mM thymidine, washed twice with PBS and released in fresh medium for 9 h with or without CDT. Cells were treated again with 2 mM thymidine for 17 h to be synchronized in early S, washed twice with PBS and released in fresh medium for 1 h before adding 5-ethynyl-2′-deoxyuridine (EdU; 5 μM) for 1 h. After two washes in PBS, cells were released in fresh medium with or without CDT for 10 h.

Cells were collected by trypsinization and fixed with 4% paraformaldehyde for 15 min at room temperature in PBS. Incorporated EdU was detected using the baseclick EdU flow-cytometry kit (Sigma, BCK-FC488) according to the manufacturer’s instructions. For immunostaining, fixed cells were permeabilized with 0.2% triton X-100 and 1% BSA in PBS for 20 min at room temperature. Antibodies against γH2AX and pH3 were incubated for 2 h in 1% BSA at room temperature. After three washes, cells were incubated with the secondary antibodies Alexa Fluor 488 anti-mouse and Alexa Fluor 594 anti-rabbit for 30 min at room temperature. Then, cells were washed three times and incubated in PBS containing DAPI (1 μg/mL) for 15 min before samples were processed using flow cytometry (MACSQuant, Miltenyi Biotec). At least 10,000 events were analyzed per sample using FlowLogic software (Miltenyi Biotec).

### Comet assay on mitotic cells

Cells were treated for 18 h with 2 mM thymidine, washed twice with PBS and released in fresh medium for 9 h. Cells were treated again with 2 mM thymidine for 17 h to be synchronized in early S, washed twice with PBS and released in fresh medium for 6 h before adding 50 ng/ml nocodazole. After 4 h, mitotic cells were collected by shake-off and exposed for 12 h to CDT. Comet assay was performed in neutral conditions using the Comet SCGE assay kit (Enzo) according to the manufacturer’s instructions. At least 60 cells were analyzed per sample using OpenComet software.

### Time-lapse imaging

HeLa cells stably expressing the chromatibody-GFP to visualize chromatin in living cells [35] were generated by TALEN insertion at AAVS1 site with co-transfection of SHDP-CMV-VHH-HA-GFP donor plasmid and AAVS1 right and left Talen plasmids (genome TALER AAVS1 safe harbor cloning kit, Genecopoeia) using TransIT-LT1 (MirusBio) according to manufacturer’s instructions. Cells were plated in 4-compartments Petri dish (CellView Greiner Bio-One). Immediately after treatment, time-lapse fluorescent microscopy was performed with a Zeiss Axio-observer inverted videomicroscope with controlled temperature (37 °C), CO2 level (5%) and humidity. Images were acquired for 90 h every 9 min with an AxioCam 506 camera (Zeiss) in two focal planes 5 µM apart from each other. Illumination has been set to 75 ms with 20% laser intensity to reduce phototoxicity. The two focal planes were flattened and 100–200 mitosis per sample were analyzed with FIJI software from 4 to 90 h post-exposure to measure the duration of mitotic phases and cell death determined morphologically (cell death was defined by morphological changes reminiscing of blebbing and cell explosion).

### Generation of cGAS knock-out HeLa cells

The CRISPR plasmid was a generous gift from J. Dupuy (Toxalim, France). The donor plasmid (pCMV-Cas9-GFP, Sigma), containing the sgRNA, Cas9 and GFP, was modified to substitute the sgRNA sequence for cGAS sgRNA 1 (5′-GCTTCCGCACGGAATGCCAGG) or cGAS sgRNA 2 (5′-CGATGGATCCCACCGAGTCT) targeting the first exon of the cGAS gene, using the Q5 site-directed mutagenesis kit (New England Biolabs) according to manufacturer’s instructions. The two plasmids bearing cGAS sgRNA1 or sgRNA2 were co-transfected in HeLa cells using TransIT-LT1 (MirusBio) according to manufacturer’s instructions. Individual clones were selected by cGAS immunofluorescence and confirmed by Western blot analyses. Two confirmed *cGAS*^*−/−*^ clones were used in this study.

### Gene expression

Total RNA was extracted with TRIzol reagent (Invitrogen, Carlsbad, CA, USA) mRNA were extracted and the quality of these samples was assessed (Agilent RNA 6000 Nano Kit Quick, Agilent Bioanalyzer 2100); RNA Integrity Number (RIN) of these mRNA was superior to 9.8.

For transcriptomic data, gene expression profiles were obtained at the GeT-TRiX facility (GénoToul, Génopole Toulouse Midi-Pyrénées, France) using Agilent SurePrint G3 Human GE v2 microarrays (8 × 60 K, design 039,494) following the manufacturer’s instructions. For each sample, Cyanine-3 (Cy3) labeled cRNA was prepared from 200 ng of total RNA using the One-Color Quick Amp Labeling kit (Agilent Technologies) according to the manufacturer's instructions, followed by Agencourt RNAClean XP (Agencourt Bioscience Corporation, Beverly, Massachusetts). Dye incorporation and cRNA yield were checked using Dropsense™ 96 UV/VIS droplet reader (Trinean, Belgium). 600 ng of Cy3-labeled cRNA were hybridized on the microarray slides following the manufacturer’s instructions. Immediately after washing, the slides were scanned on Agilent G2505C Microarray Scanner using Agilent Scan Control A.8.5.1 software and fluorescence signal extracted using Agilent Feature Extraction software v10.10.1.1 with default parameters.

For real-time quantitative polymerase chain reaction (qPCR), 2 μg RNA samples were reverse-transcribed using the High-Capacity cDNA Reverse Transcription Kit (Applied Biosystems). qPCR was performed using the Power SYBR® Green PCR Master Mix and an ABI Prism 7300 Sequence Detection System instrument and software (Applied Biosystems). Sequences of the primers are listed in supplementary Table 1. All samples were run in triplicate. qPCR data were normalized to TBP1 mRNA levels and analyzed with LinRegPCR.v2015.3.

### ELISA assays

ELISA assay against human IL-6 (E-EL-H0102) was purchased from Elabscience, mouse IL-6 (DY406) from R&D Systems and mouse TNFα (3511-1H-20) from Mabtech. All assays were performed on cell culture supernatant according to the manufacturer’s instructions.

### Statistical analysis

Microarray data were analyzed using R and Bioconductor packages [58]. Raw data (median signal intensity) were filtered, log_2_ transformed, and normalized using quantile method [59]. A model was fitted using the limma lmFit function [60]. Pair-wise comparisons between biological conditions were applied using specific contrasts. A correction for multiple testing was applied using Benjamini–Hochberg procedure to control the False Discovery Rate (FDR). Probes with FDR ≤ 0.05 were considered to be differentially expressed between conditions. Hierarchical clustering was applied to the samples and the differentially expressed probes using 1-Pearson correlation coefficient as distance and Ward’s criterion for agglomeration.

Other statistical analyses were assessed using Prism 8 software (GraphPad). Differential effects were analyzed by one-way or two-way analysis of variance (ANOVA) followed by appropriate post hoc tests (Sidak, Tukey or Dunnett). A *p* value < 0.05 was considered significant (*p < 0.05; **p < 0.01; ***p < 0.001; ****p < 0.0001).

## Results

### CDT induces a proinflammatory signature related to type I interferon

**Fig. 1:**
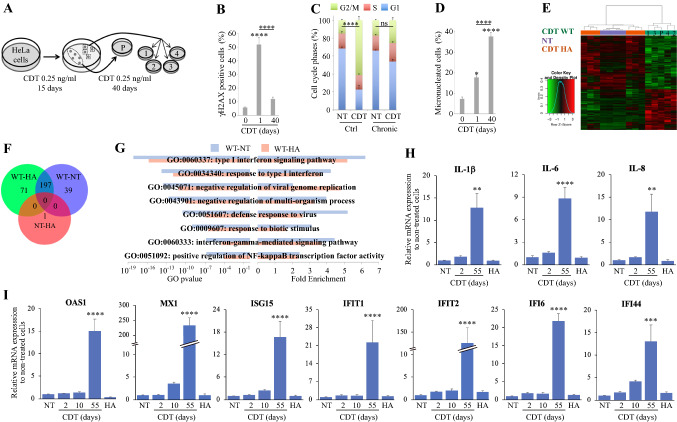
Chronic exposure to CDT induces a type I IFN response. **a** Schematic of the selection procedure of HeLa cells chronically exposed to CDT. Individual clones (1–4) and a multiclonal cell population (P cells) were selected. **b** HeLa cells exposed for 24 h to CDT 0.25 ng/ml and P cells from **a** were analyzed by immunofluorescence microscopy to quantify the frequency of γH2AX-positive cells. Data represent the mean ± SEM of at least 3 independent experiments. Statistics were calculated by one-way ANOVA followed by Tukey’s multiple comparison test. **c** HeLa cells and the P cells from **a** were exposed to CDT 2.5 ng/ml for 24 h and subjected to cell cycle analyzes by flow-cytometry. Data represent the mean ± SEM of at least three independent experiments. Statistics (only G2/M) were calculated by two-way ANOVA followed by Sidak’s multiple comparison test. **d** HeLa cells treated as in (**b**) were analyzed by immunofluorescence microscopy to quantify the frequency of micronucleated cells. Data represent the mean ± SEM of at least three independent experiments. Statistics were calculated by one-way ANOVA followed by Tukey’s multiple comparison test. (**e**–**g**) Non-treated (NT) HeLa cells (two individual clones and two samples of non-selected cells), or cells from **a** exposed to wild-type CDT (CDT WT, four individual clones and one sample of P cells) or the catalytic mutant H153A (CDT HA, 5 individual clones and one sample of P cells) were compared after microarray gene expression analysis. Heatmap **e** shows the significantly altered genes expression (FDR < 0.05 and fold change > 1), and Venn diagram **f** shows overlap between the most upregulated genes (FDR < 0.05 and fold change > 3). Graphs **g** represent the eight most significant upregulated biological processes sorted according to adjusted *p* value obtained from a hypergeometric enrichment test on Gene Ontology (GO) processes. **h**, **i** HeLa cells non-treated (NT) or exposed to CDT WT 0.25 ng/ml for 2 days, and cells from **a** exposed to CDT WT (three individual clones and one sample of P cells) or HA (two individual clones and two samples of P cells) were collected and the mRNA level of indicated genes was analyzed by RT-qPCR. Data represent the mean ± SEM of at least three independent experiments. Statistics were calculated by one-way ANOVA followed by Dunnet’s multiple comparison test

To determine whether chronic exposition to CDT induces an inflammatory response in the host, a model human cancer cell line (HeLa) was chronically exposed to 0.25 ng/ml of *E. col* CDT, inducing more than 95% cell death after 10 days (Supp Fig. 1). The surviving fraction was cultured for 40 more days in presence of CDT and individual clones were selected as well as a pool of resistant cells (Fig. 1a). Compared to a short-term exposure, cells chronically treated to CDT (55 days total) do not show significant increase of γH2AX level, used as a marker of DNA damage signaling (Fig. 1b). In addition, these cells were unresponsive to the CDT-mediated G2/M checkpoint (Fig. 1c), suggesting an adaptation to the CDT toxin. However, chronically exposed cells exhibit a higher proportion of micronucleated cells, indicative of important chromosomal instability (Fig. 1d). These cells were subjected to transcriptomic analyses and compared to two control groups, i.e. cells without treatment or chronically exposed to the CDT catalytic dead mutant, bearing the H153A substitution on CdtB (H153A), which cannot induce DNA damage nor activate DDR [33, 34]. As depicted in the heatmap resuming expression profile of 9703 significantly regulated genes between these three conditions, individual clones and the pool of cells chronically exposed to active wild-type (WT) CDT share a common transcriptional adaptation, whereas the two control groups (non-treated and treated with CDT H153A) cannot be distinguished (Fig. 1e). The majority of the most upregulated genes, when comparing the three groups, depends on the catalytic activity of CDT rather than the presence of the toxin solely (Fig. 1f). Strikingly, the most upregulated biological processes in cells chronically exposed to WT CDT mainly rely on immune responses, more particularly to type I IFN signaling (Fig. 1g). To confirm that CDT exposure elicits a proinflammatory signature, mRNA expression level of IL-1β, IL-6, and IL-8 cytokines was determined after only 2 days of CDT WT or after repeated treatment with CDT WT or H153A during 55 days (Fig. 1h). In cells chronically exposed to CDT WT, proinflammatory cytokines mRNA expression level statistically increases, around tenfold compared to non-treated cells. This depends on the CdtB catalytic activity, as H153A mutation abolishes expression profile modification. In the same way, short exposure to 0.25 ng/ml of CDT WT during 2 days does not significantly alter cytokines expression level. We next tested a panel of ISGs related to type I IFN response (*OAS1*, *MX1*, *ISG15*, *IFIT1*, *IFIT2*, *IFI6* and *IFI44*). While ISGs expression level does not significantly increase after 2 or 10 days of CDT treatment, a chronic 55 days exposure strongly enhances their expression with CDT WT but not H153A (Fig. 1i). Taken together, these results show that cells chronically exposed to CDT accumulate MN and modulate their immune response through increase of proinflammatory response and type I IFN signaling.

### cGAS binds CDT-mediated micronuclei and promotes type I IFN response

**Fig. 2:**
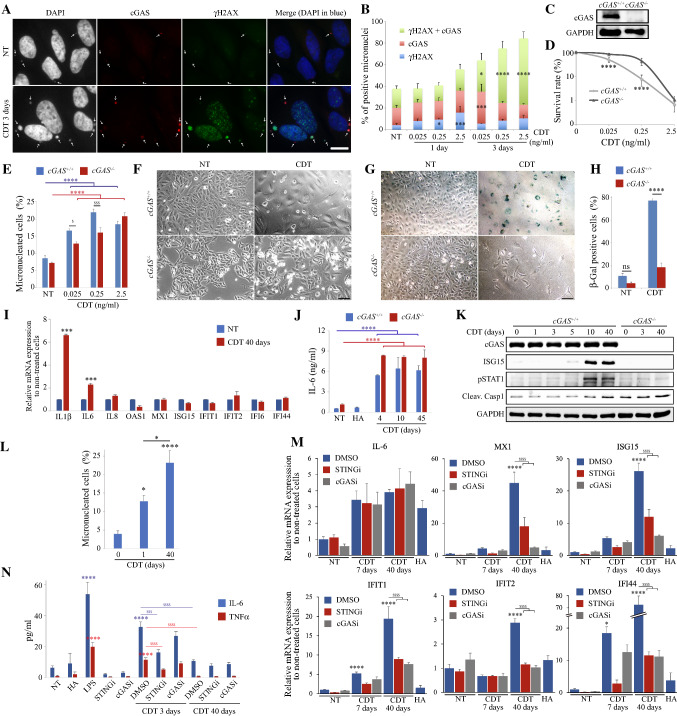
CDT-induced micronuclei promote a cGAS-dependent type I IFN response. **a**, **b** HeLa cells were exposed for 1 or 3 days to CDT and analyzed by immunofluorescence microscopy with antibodies against cGAS and γH2AX. Representative images (**a**) and quantification of micronuclei positive for cGAS, γH2AX, or double positive for cGAS + γH2AX (**b**) are shown. White arrows indicate micronucleus localization (**a**). Scale bar = 20 μm. **c** Soluble fractions of *cGAS*^+*/*+^ and *cGAS*^*−/−*^ HeLa cells were analyzed by Western blotting. **d** *cGAS*^+*/*+^ and *cGAS*^*−/−*^ HeLa cells were exposed to CDT and subjected to colony formation assay. **e** *cGAS*^+*/*+^ and *cGAS*^*−/−*^ cells were exposed for 24 h to CDT and analyzed by immunofluorescence microscopy to quantify the frequency of micronucleated cells. **f**–**h** *cGAS*^+*/*+^ and *cGAS*^*−/−*^ HeLa cells were non-treated (NT) or exposed to CDT 2.5 ng/ml for 90 h (CDT) and analyzed by light microscopy without (**f**) or with β-Gal staining (**g**, **h**). Scale bars = 100 μm. Quantification of β-Gal-positive cells is shown (**h**). **i** *cGAS*^*−/−*^ HeLa cells were non-treated (NT) or exposed to CDT 0.25 ng/ml for 40 days and the mRNA level of the indicated genes were analyzed by RT-qPCR. **j** IL-6 concentration was determined by ELISA in the culture supernatant of *cGAS*^+*/*+^ and *cGAS*^*−/−*^ HeLa cells non-treated (NT) or exposed to CDT 0.25 ng/ml for the indicated times or to the H153A CDT mutant for 45 days (HA). **k** *cGAS*^+*/*+^ and *cGAS*^*−/−*^ HeLa cells were exposed to CDT 0.25 ng/ml for the indicated times and soluble fractions were analyzed by Western blotting. **l** MEFs were exposed to CDT 7.5 ng/ml for 0, 1 or 40 days and analyzed by immunofluorescence microscopy to quantify the frequency of micronucleated cells. **m**, **n** MEFs were exposed with or without STINGi or cGASi to CDT 7.5 ng/ml for the indicated times or to the H153A CDT mutant for 40 days (HA). The mRNA level of the indicated genes was analyzed by RT-qPCR (**m**) and the IL-6 or TNFα production in the culture supernatant were determined by ELISA (**n**). For ELISA assays, a 2 days exposure to LPS 100 ng/ml was used as positive control. **b**, **d**, **e**, **h, i, j**, **l**–**n** Data represent the mean ± SEM of at least three independent experiments. Statistics were calculated by one-way (**i**, **l**–**n)**  or two-way (**b**, **d**, **e**, **h**, **j**) ANOVA followed by Sidak’s (**b**, **d**, **e**, **h**, **i**, **m**, **n**) or Tukey’s (**j**, **l**) multiple comparison test

MN recognition by cGAS triggers innate immune activation related to type I IFN signature [27, 28]. We thus questioned whether cGAS could bind to CDT-induced MN (Fig. 2a) and examined the proportion of MN recognized by cGAS or stained with a γH2AX antibody. After 24 h of CDT exposure, a dose-dependent increase of γH2AX-positive MN can be observed (Fig. 2b). In contrast, the proportion of cGAS-positive MN increases only after 72 h, with MN progressively accumulating γH2AX staining by increasing CDT concentration. Therefore, cGAS recognizes CDT-induced MN, but this binding is delayed in time.

To better understand the role of cGAS in response to CDT injury, cGAS knockout HeLa cells (*cGAS*^*−/−*^) were generated (Fig. 2c). *cGAS*^*−/−*^ cells are more resistant to low CDT concentrations (0.025 and 0.25 ng/ml) than their WT cGAS counterpart (Fig. 2d) and accumulate less MN (Fig. 2e). Moreover, cGAS-deficient cells exhibit less cell distention (Fig. 2f). In the same way, the increase of β-Gal staining, a marker of cellular senescence, is less important in cGAS-depleted cells (Fig. 2g, h). These results indicate that cGAS might regulate MN formation and proliferation of HeLa cells exposed to CDT, at least partly by promoting senescence.

Then, we examined the mRNA expression level of proinflammatory cytokines and ISGs in *cGAS*^*−/−*^ HeLa cells chronically exposed to CDT (Fig. 2i). Only IL-1β and IL-6 expression was significantly upregulated after 40 days of CDT exposure, whereas ISGs were not affected. Although IL-6 mRNA upregulation is less marked compared to *cGAS*^+*/*+^ cells (Fig. 1h), the increased concentration of secreted IL-6 cytokine is similar with or without cGAS, all along CDT exposure (Fig. 2j). Of note, only an active CDT toxin can stimulate IL-6 production, as revealed by chronic exposure to the H153 mutant. Thus, cGAS does not seem to impact IL-6 production during chronic exposure to CDT. Conversely, we found that ISG15 protein level was only enhanced in presence of cGAS, and could not be observed during the first 5 days of CDT treatment (Fig. 2k). Furthermore, this late ISG15 increase was accompanied by STAT1 phosphorylation at Y701 (pSTAT1), a surrogate marker for type I IFN signaling [27], that is not induced in *cGAS*^*−/−*^ cells. Finally, we monitored inflammasome activation through Caspase-1 self-cleavage and did not observe any defect in *cGAS*^*−/−*^ cells, but rather an increased Caspase-1 cleavage. Altogether, these results establish that CDT chronic exposure in HeLa cells triggers a cGAS-dependent type I IFN signaling that is delayed in time and a cGAS-independent proinflammatory response.

To confirm that these immunomodulatory effects represent a general response to CDT, these experiments were reproduced on *cGAS*^+*/*+^ and *cGAS*^*−/−*^ HeLa cells treated with CDT from *H. duc* (*H. duc* CDT). Both cell lines exhibited similar enhanced proportion of micronucleated cells after a chronic exposure to *H. duc* CDT (Supp Fig. 2a). In contrast to *E. col* CDT, the proinflammatory response was higher in cGAS-defective cells than their wild-type counterpart (Supp Fig. 2b, c). Conversely, ISGs gene induction in response to *H. duc* CDT is less effective than with *E. col* CDT but still cGAS-dependent (Supp Fig. 2d). Finally, this was confirmed at the protein level for ISG15 (Supp Fig. 2e).

Similar experiments were also conducted on primary MEFs. MEFs chronically exposed to CDT still present MN after 40 days (Fig. 2l). These cells were co-exposed or not with the STING inhibitor H-151 (STINGi) or the cGAS inhibitor RU.521 (cGASi) and analyzed for IL-6 and ISGs mRNA expression (Fig. 2m). After 7 or 40 days of CDT, IL-6 mRNA expression shows a slight increase that is not statistically significant and is not affected by STING or cGAS inhibition. On the contrary, ISGs expression was enhanced after 40 days but not 7 days of CDT treatment, reaching up to 72.7-fold increase for IFI44 compared to non-treated cells. ISGs gene overexpression in response to CDT was not found upon exposure to the inactive H153 CDT mutant, and was impaired by STING or cGAS inhibition. This confirms the crucial role of cGAS-STING axis in CDT-induced type I IFN signaling. Finally, IL-6 and TNFα production was elevated in response to 3 days of CDT exposure in a STING-dependent manner (Fig. 2n). However, IL-6 and TNFα concentrations dropped back to the basal level after 40 days, indicating that the proinflammatory signature elicited by CDT is not maintained upon chronic exposure.

### CDT-exposed cells reach mitosis despite active G2 cell cycle checkpoint

**Fig. 3:**
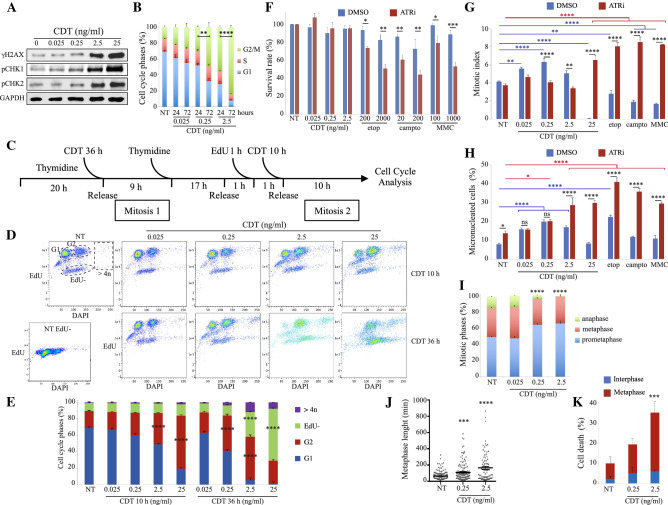
CDT-exposed cells do not block at G2 during the first cell cycle and show mitotic defects. **a** HeLa cells were exposed for 24 h to CDT and soluble fractions were analyzed by Western blot with the indicated antibodies. **b** HeLa cells were exposed for 24 or 72 h to CDT and subjected to cell cycle analyzes by flow-cytometry. Statistics were calculated only for G2/M. **c**–**e** Cell cycle analysis of synchronized HeLa cells after CDT treatments and EdU pulse labeling. Experimental workflow is presented in (**c**). The timing of each treatment between wash and release in fresh medium is indicated below the timeline. During the synchronization procedure, cells were treated with CDT before the first mitotic division (CDT 36 h) or before the second mitotic division (CDT 10 h). Graphs of the cell cycle profiles from one representative experiment are presented in (**d**). Control corresponding to non-treated cells without EdU pulse labeling is shown (NT EdU-). Identification of the different cell populations is shown on the non-treated condition (NT): EdU-positive cells in G1 or G2 phase, EdU-negative cells (EdU-) and cells with more than 4*n* chromosomes (> 4*n*). Quantification of the cell populations is shown in (**e**). **f** HeLa cells were exposed for 24 h to CDT, etop, campto or MMC with or without ATRi and subjected to CellTiter-Glo viability assay. **g**, **h** HeLa cells were exposed for 24 h to CDT, etop 200 nM, campto 20 nM or MMC 100 nM with or without ATRi. Mitotic index (**g**) and the frequency of cells with MN (**h**) were quantified. **i** HeLa cells were exposed for 24 h to CDT and the proportion of prometaphase, metaphase or anaphase were quantified. Statistics were calculated only for anaphase. **j**, **k** HeLa cells stably expressing the chromatibody-GFP [35] were exposed to CDT and analyzed by time-laps fluorescence imaging. Graph from (**j**) show the average time duration of metaphase to anaphase transition ± SEM of one representative upon three independent experiments. **b, e**–**i**, **k** Data represent the mean ± SEM of at least three independent experiments. Statistics were calculated by one-way (**e**, **i**–**k**) or two-way (**b**, **f**–**h**) ANOVA followed by Sidak’s (**b**, **f**–**h**) or Dunnett’s (**e**, **i**–**k**) multiple comparison test

As cGAS-mediated type I IFN response depends on MN recognition, we next asked whether MN formation is the direct consequence of CDT intoxication. Previous studies from our lab and others showed that CDT-induced DNA damage activate the G2/M checkpoint [21]. However, G2/M checkpoint arrest is inconsistent with MN formation that requires mitosis completion. To explain the accumulation of MN following CDT treatment, we first monitored DNA damage markers. As DNA damage checkpoints rely on DDR activation, phosphorylation of H2AX at S139 (γH2AX), CHK1 at S345 (pCHK1) and CHK2 at T68 (pCHK2) were measured after a 24 h treatment with CDT (Fig. 3a). Strong DDR activation is only observed at high concentration of CDT (2.5 and 25 ng/ml). This result supports that at low concentrations, the proliferation defects induced by CDT (Supp Fig. 1) is unlikely the consequence of a rapid DDR activation and immediate checkpoint-induced cell cycle arrest. Indeed, the CDT-mediated cell cycle arrest significantly increases from 24 to 72 h (Fig. 3b), implying that at least a part of CDT-exposed cells reach mitosis before to block their cell cycle during the next rounds of cell division. In contrast, exposure to etoposide (etop), camptothecin (campto) or mitomycin C (MMC), three other genotoxic compounds, induces a rapid and stable cell cycle block over time (Supp Fig. 3). To demonstrate that CDT-exposed cells can complete a first mitotic division prior efficient cell cycle arrest at the next G2, Hela cells were pulse-labeled with EdU in early S phase after a double thymidine cell synchronization, either being exposed to CDT after the first (“CDT 36 h” condition) or the second (“CDT 10 h” condition) thymidine bloc (Fig. 3c). In absence of CDT, around 90% of cells incorporated EdU, demonstrating that cell synchronization was effective (Fig. 3d). Moreover, 68.5% of EdU-positive cells proceeded to G1, implying they passed through G2 and mitosis. For the “CDT 10 h” condition, cells exposed to 0.025 and 0.25 ng/ml of CDT do not show any significant increase of the G2 cell population, demonstrating they did not activate the G2 checkpoint at the first cell cycle during CDT treatment (Fig. 3e). However, cells exposed to 0.25 ng/ml of CDT during the previous cell cycle (“CDT 36 h” condition) exhibit 42.8% of G2 cells compared to 20.8% without CDT, indicating that the G2 checkpoint was activated in response to CDT after one cell division. In contrast, a dose-dependent G2 arrest of the EdU-positive population is observed from 2.5 ng/ml of CDT for the “CDT 10 h” condition. This demonstrate that CDT-treated cells arrest in the first G2 phase only at high doses. Moreover, a significant increase of EdU-negative cells occurs in the “CDT 36 h” condition at 2.5 and 25 ng/ml of CDT, representing cells that did not reached S phase at the time of EdU labeling, therefore, cells that were arrested at the previous G2. To conclude, these results represent a direct evidence that CDT-exposed cells pass through mitosis before blocking at the next G2, except at highest concentrations.

To test whether DDR does not effectively abrogate cell proliferation during early phase of CDT intoxication, cells were co-exposed during 24 h to CDT and the ATR inhibitor VE-821 (ATRi), given that the G2/M checkpoint mostly depends on ATR rather than ATM under these conditions (Supp Fig. 4). Contrary to other genotoxic treatments, ATR inactivation does not sensitize HeLa cells to CDT during the first 24 h of exposure (Fig. 3f), further supporting a minor G2 checkpoint activation during early phase of CDT intoxication. Moreover, while exposure to control genotoxicants or high CDT concentration (25 ng/ml) block mitotic entry in an ATR-dependent manner, indicative of active G2 checkpoint, lowest CDT concentrations significantly increase the mitotic index, confirming that cells do progress through mitosis (Fig. 3g). This result demonstrates that ATR is only crucial after high treatment with CDT to protect cells from mitotic catastrophe by inducing a G2/M arrest, at least during the first 24 h of exposure. Finally, in contrast to high CDT concentrations or DNA damaging agents, low CDT concentrations (0.025 and 0.25 ng/ml) induce MN formation that is not aggravated by the presence of ATRi (Fig. 3h). Altogether, these data demonstrate that except for high concentrations, CDT exposure allows mitotic entry and MN generation, despite the presence of active cell cycle checkpoints.

### CDT induces mitotic delay and cell death

The increased mitotic index observed in CDT-exposed cells (Fig. 3g) is accompanied by a dose-dependent diminution of the anaphase population (Fig. 3i). To gain insight into the mitotic phenotype of CDT-treated cells, live-cell imaging has been performed on HeLa cells stably expressing the chromatibody fused to GFP, enabling real-time chromatin visualization [35]. When measuring the timing needed to complete metaphase, we found that unperturbed mitosis takes an average of 64 min that significantly increases to 109 and 164 min after treatment with 0.25 ng/ml and 2.5 ng/ml of CDT, respectively (Fig. 3j). Moreover, monitoring cell death during the course of live imaging revealed that an important fraction of CDT-exposed cells preferentially dies at metaphase (Fig. 3k). In conclusion, mitotic cells are particularly affected during CDT intoxication, as evidenced by a prolonged metaphase duration that eventually results in cell death.

### CDT-exposed cells experience DNA damage at mitosis

**Fig. 4:**
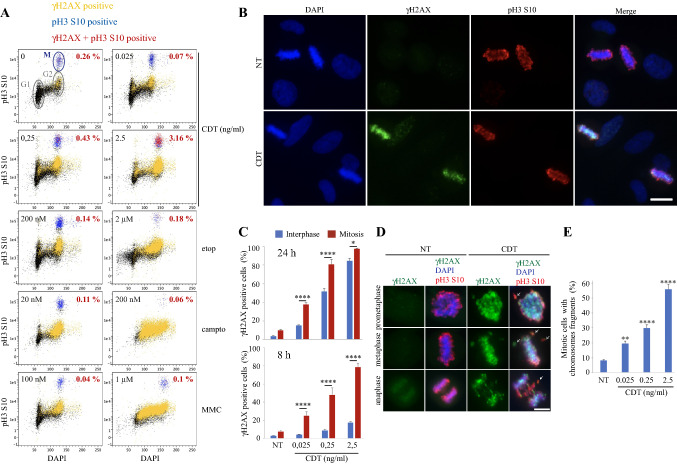
CDT-exposed cells accumulate DNA damage at mitosis. **a** HeLa cells were exposed for 24 h to CDT, etop, campto or MMC, immunostained with antibodies directed against γH2AX and pH3 and subjected to cell cycle analyzes by flow-cytometry. Graphs of the cell cycle profiles from one representative upon two independent experiments show unstained cells (black), or with γH2AX (yellow), pH3 (M for mitosis, blue) or both staining (red). Percentage in red indicate the proportion of γH2AX + pH3-positive cells. **b**, **c** HeLa cells were exposed to CDT for 24 h or 8 h, and analyzed by immunofluorescence microscopy with antibodies directed against γH2AX and pH3. Representative images (**b**) and quantification (**c**) are shown. Scale bar = 20 μm. Data represent the mean ± SEM of at least three independent experiments. Statistics were calculated by two-way ANOVA followed by Sidak’s multiple comparison test. **d**, **e** HeLa cells were exposed for 24 h to CDT and analyzed by immunofluorescence microscopy with antibodies directed against γH2AX and pH3. Representative images of mitotic cells in prometaphase, metaphase or anaphase (**d**) and quantification of chromosome fragments in prometaphase and metaphase (**e**) are shown. Scale bar = 10 μm. White arrows indicate chromosome fragments. Data represent the mean ± SEM of at least three independent experiments. Statistics were calculated by one-way ANOVA followed by Dunnett’s multiple comparison test

To better understand the relationship between CDT-mediated DNA damage and cell cycle defects, cell cycle analyses were conducted after immunostaining with antibodies directed against γH2AX and H3 histone phosphorylated at S10 (pH3) to identify mitotic cells. Cells treated with CDT for 24 h present a dose-dependent augmentation of pH3 and γH2AX positive cells, representing a 12-fold increase at 2.5 ng/ml of CDT compared to control cells (Fig. 4a). In contrast, after exposure with moderate concentration of control genotoxic compounds, only cells without γH2AX staining do progress to mitosis. Thus, CDT-exposed cells progress through mitosis with damaged DNA, representing a unique feature over other genotoxic insult. Strikingly, asynchronous cells exposed to CDT display an intense γH2AX signal in mitosis compared to interphase (Fig. 4b). This staining is clearly distinguishable from the basal DNA damage-independent γH2AX signal described in unchallenged mitotic cells [36], that is diffuse all along the condensed chromosomes from prometaphase to anaphase, or from few γH2AX foci observed in mitotic cells exposed to other DNA damaging agents (Supp Fig. 5). The huge γH2AX increase at mitosis is observed with CDT from other bacterial origins or with other cell lines (Supp Fig. 6), thus representing a general cellular response to CDT. Moreover, the fraction of γH2AX-positive cells is more important in mitosis compared to interphase, after 24 h or even a shorter incubation of 8 h with CDT (Fig. 4c), demonstrating that mitotic cells represent the first population to be damaged during the course of CDT treatment. The strong γH2AX signal after CDT can be observed all along the mitotic phases (Fig. 4d). Finally, CDT exposure induces a dose-dependent increase of chromosome fragments that does not properly align during metaphase or segregate at anaphase (Fig. 4e), therefore, explaining the high level of MN observed after CDT treatment.

### CDT induces DNA double-strand breaks during mitosis

**Fig. 5:**
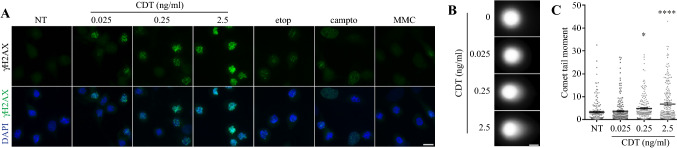
CDT induces DNA damage during mitosis. **a** HeLa cells were exposed for 22 h to nocodazole, with CDT, etop 200 nM, campto 20 nM or MMC 100 nM during the last 6 h and analyzed by immunofluorescence microscopy with an antibody directed against γH2AX. Images of one representative upon four different experiments experiment are shown. Scale bar = 20 μm. **b**, **c** HeLa cells were synchronized in mitosis, exposed for 12 h to CDT and analyzed by neutral comet assay. Representative images (**b**) and quantification of individual cells tail moment (**c**) are shown. Scale bar = 20 μm. Data represent the mean ± SEM of three independent experiments. Statistics were calculated by one-way ANOVA followed by Dunnett’s multiple comparison test

To exclude the possibility that mitotic γH2AX signal originates from DNA damage induced before mitotic entry, HeLa cells were enriched in mitosis by a 22 h nocodazole block and then co-exposed during the last 6 h to CDT or genotoxic control agents. Similar to observations made on asynchronous cells, cells treated with CDT during mitosis exhibit a strong γH2AX level compared to etop, campto or MMC (Fig. 5a). To confirm that the γH2AX level increase in mitosis depends on DSB induction, cells arrested in mitosis were exposed to CDT before to be subjected to neutral comet assay (Fig. 5b). Mitotic cells treated with 0.25 or 2.5 ng/ml of CDT show a significant increase of comet tail moment (Fig. 5c). Taken together, these data demonstrate that CDT induces DSB during mitosis leading to chromosome fragmentation and missegregation.

### Immortalized normal colonic epithelial cells chronically exposed to CDT display an altered type I IFN response associated to cGAS protein loss

**Fig. 6:**
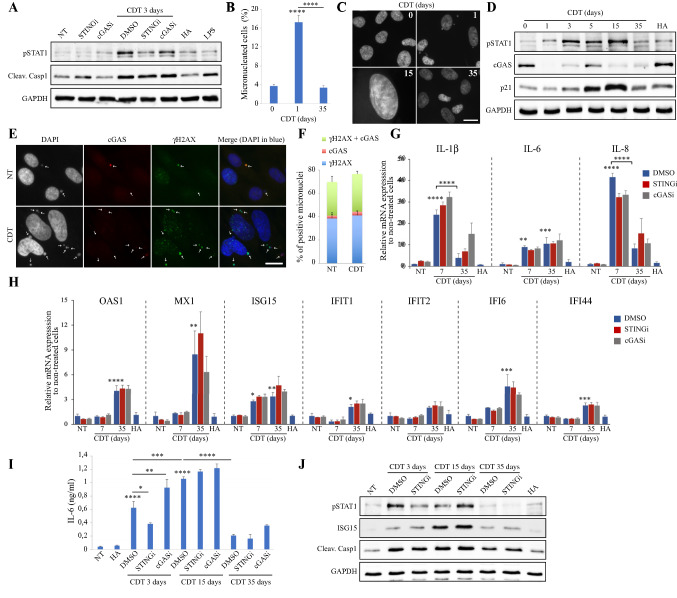
CDT downregulates cGAS in non-transformed HCEC cells. **a** HCEC cells were non-treated (NT), or exposed with or without STINGi or cGASi for 3 days to 0.25 ng/ml of CDT or the H153A CDT mutant (HA), or to LPS 100 ng/ml for 2 days. Soluble fractions were analyzed by Western blotting with the indicated antibodies. **b** HCEC cells were exposed to CDT 0.25 ng/ml for the indicated times and analyzed by fluorescence microscopy to quantify the frequency of micronucleated cells. **c** Representative images of HCEC cells treated as in (**b**). Scale bar = 20 μm. **d** HCEC cells were non-treated (NT), or exposed for the indicated time to 0.25 ng/ml of CDT or the H153A CDT mutant for 10 days (HA). Soluble fractions were analyzed by Western blotting with the indicated antibodies. **e**, **f** HCEC cells were non-treated (NT) or exposed for 3 days to CDT and analyzed by immunofluorescence microscopy with antibodies against cGAS and γH2AX. Representative images (**e**) and quantification of micronuclei positive for cGAS, γH2AX, or double positive for cGAS + γH2AX (**f**) are shown. White arrows indicate micronucleus localization (**e**). Scale bar = 20 μm. **g**, **h** HCEC cells were exposed with or without STINGi or cGASi to CDT 0.25 ng/ml for the indicated times or to the H153A CDT mutant for 35 days (HA). The mRNA level of the indicated genes was analyzed by RT-qPCR. **i** IL-6 concentration was determined by ELISA in the culture supernatant of HCEC cells non-treated (NT) or exposed with or without STINGi or cGASi to CDT 0.25 ng/ml for the indicated times or to the H153A CDT mutant for 35 days (HA). **j** HCEC cells were treated as in (**i**). Soluble fractions were analyzed by Western blotting with the indicated antibodies. **b**, **g**–**i** Data represent the mean ± SEM of at least three independent experiments. Statistics were calculated by one-way ANOVA followed by Tukey’s multiple comparison test

We next assessed whether non-transformed colonic cells exhibit similar immune response to CDT genotoxic activity. Immortalized normal human colonic epithelial cells (HCECs), previously shown to be susceptible to CDT intoxication [37], present similar mitotic defects than HeLa cells (Supp Fig. 7). HCECs were exposed for 3 days to 0.25 ng/ml of CDT, with or without STING or cGAS inhibitors, and type I IFN signaling or inflammasome activation were assessed through pSTAT1 and cleaved Caspase-1, respectively (Fig. 6a). Contrary to the inactive H153A toxin, CDT induced an increase in pSTAT1 and cleaved Caspase-1 level. Cleaved Caspase-1 also increased in response to LPS and was not dependent on STING or cGAS activity. Intriguingly, STINGi but not cGASi could decrease pSTAT1 in CDT-exposed cells. To further analyze HCECs behavior, we measured the proportion of MN-containing cells and found that CDT-induced MN formation observed after 1 day is lost after 35 days (Fig. 6b). During the course of long-term CDT treatment, HCECs enter a senescence state associated to cell distention that is independent on STING or cGAS activities (data not shown) and maintained for at least 15 days (Fig. 6c). At these time points, p21 and pSTAT1 level increased with CDT but not with the H153A inactive mutant (Fig. 6d). These cells then escape from senescence and restart to proliferate with a basal level of micronucleated cells as seen after 35 days of chronic exposure, while p21 and pSTAT1 decreased. Strikingly, the level of cGAS protein was continuously disrupted all along the chronic exposure to CDT but not the inactive mutant. Furthermore, a low cGAS signal is observed in MN after 3 days of CDT treatment (Fig. 6e), and the proportion of cGAS-positive MN is not enhanced compared to untreated cells (Fig. 6f). Taken together, these results suggest that CDT exposure impedes cGAS-mediated response in HCECs by affecting cGAS protein stability and MN recognition. Moreover, the low pSTAT1 and MN levels of HCECs that escaped senescence also imply that the type I IFN response is not maintained during long-time exposure to CDT. We next monitored mRNA expression level of proinflammatory cytokines and ISGs in HCECs after 7 days (senescence state) or 35 days of CDT exposure (Fig. 6g, h). As observed in MEFs, proinflammatory cytokines mRNA level increased after 7 days independently of STING or cGAS activities, but decreased after 35 days with CDT. ISGs mRNA expression only shows a slight increase compared to HeLa or MEFs (Figs. 1i and 2m), and was not affected by STING or cGAS inhibitors, confirming that the cGAS-STING axis is not activated in HCECs after chronic exposure to CDT. Finally, kinetics of proinflammatory cytokine mRNA expression is reflected by the low IL-6 concentration measured in the culture medium after 35 days of CDT treatment compared to 15 days (Fig. 6i). Similar to MEFs, IL-6 increase at 3 days partially depends on STING, but not after 15 days. In the same way, STINGi decreases pSTAT1 level induced by 3 days of CDT exposure but not after 15 days (Fig. 6j). ISG15 protein increased until 15 days but decreased at 35 days of CDT treatment, and the CDT-induced cleavage of Caspase-1 also diminishes at latter time point. To conclude, proinflammatory and type I IFN responses are poorly activated in HCECs during long-time exposure to CDT, which is probably correlated to cGAS protein downregulation and loss of MN induction after senescence escape.

## Discussion

Cell distention and other features of CDT exposure, namely cell cycle arrest and proinflammatory cytokines expression, have been associated to cellular senescence [25]. However, inflammatory response of cells that escaped senescence after long-time exposure to CDT has never been addressed so far. Our study now establishes a direct link between CDT-mediated type I IFN response during chronic exposure and the cytosolic DNA sensor cGAS recognizing MN that, at least in part, may arise from DNA damage induced during mitosis.

Mitosis coordinates the proper segregation of sister chromatids to ensure faithful transmission of equal genetic material to the next cell generation. On the other side, DSB repair is inhibited during mitosis, through the phosphorylation of several DDR factors by CDK1 and PLK1 kinases, noticeably to prevent telomere fusion [38]. Therefore, controlling chromosome integrity prior separation is performed at the previous G2 phase and implies cell cycle arrest and DNA repair to impede transition to mitosis with damaged DNA [39]. As duration of M phase is short and constant [40], only a very minor part of cells from an asynchronous population may suffer DNA damage at mitosis. The data presented here depict an atypical scenario with CDT-treated cells being able to reach mitosis despite continuous DNA damage induction. Contrary to ionizing radiations that instantaneously induce massive DNA damage, or to chemicals and metabolites from pathogens with limited stability or capacity to induce repeated lesions, the CDT catalytic subunit CdtB may exert unceasing genotoxic attacks, given its nuclease activity remaining active for at least 48 h after cellular internalization [24, 34]. CDT is the only characterized encoded toxin from mammalian pathogens that has evolved to generate DSBs in host genomic DNA [41]. This continuous nuclease activity is opposed to the host DNA repair machinery, implying that under a certain threshold, CDT-induced DNA damage do not effectively activate DDR and, therefore, allows G2/M transition. In this context, previous studies used most of the time CDT concentration around 1 μg/ml and beyond, resulting in substantial cell cycle arrest at the G2 checkpoint. Similar effects have been observed here in HeLa cells with 25 ng/ml of CDT within the first 24 h of exposure, whereas a 1000-fold lower concentration does not strongly activate DDR nor alter cell cycle, but still induces DNA damage, chromosome fragmentation and a delay at mitosis followed by increased MN formation and eventually viability loss. Currently, the physiological concentration of CDT to which cells are exposed during a natural infection is unknown [12], but one may expect that the cellular effects caused by moderate concentrations of toxin used in this study should be at least as relevant than analyzing highest doses.

We demonstrate here that once entered in mitosis, CDT-treated cells experience massive DNA damage, as evidenced by the intense mitotic γH2AX staining. In contrast, interphasic cells exhibit much lowest γH2AX signal. We speculate that this difference might be a consequence of DNA repair inhibition during mitosis [38], resulting in DNA damage accumulation and thus continuous H2AX phosphorylation. High mitotic γH2AX staining is observed in several human cell lines, with CDT toxins from various bacterial origins. This indicates that inflicting DNA lesion during mitosis is a general mode of action of CDT in proliferative cells. The γH2AX signal in mitosis is correlated to a dose-dependent increase of mitotic cells with chromosome fragments, as revealed by microscopic observation or neutral comet assay. These DNA damage impose a dose-dependent mitotic delay at metaphase, similar to cells depleted for the mitotic resolvase GEN1 [42, 43]. More generally, mitotic duration is governed by the capacity of cells to dissolve or resolve Hollyday junctions [44], questioning on CdtB abilities to target homologous recombination intermediates that are processed later in mitosis.

We speculate that the progressive accumulation of DNA damage, during at least 48 h, in interphasic CDT-exposed cells [33] may partly be a consequence of unrepaired DNA lesions transmitted from the previous cell division. In comparison, control genotoxic compounds induce only few mitotic DSBs at selected concentrations that only partially block cells in G2, in an ATR-dependent manner. Etoposide at 10 μM has been shown to induce important DNA damage on mitotic HeLa cells, however, synchronization in mitosis is required to ensure that an important proportion of cells is affected [45]. Alternatively, HT-29 cells arrested in G2 after a 25 nM treatment of camptothecin, a concentration that is comparable to the present study, finally reach mitosis 48 h post-treatment with highly damaged chromatin [46]. Such recovery from G2 arrest in the presence of DNA damage, referred to as checkpoint adaptation, has been observed with many DNA damaging agents and give rise to important MN formation in post-mitotic cells [47]. Therefore, CDT exposure globally recapitulates the phenotype of cells subjected to checkpoint adaptation, but with more rapid kinetics due to a weak ATR-dependent G2 checkpoint activation.

The cGAS-STING axis activates IRF3 and NF-κB, two transcriptional inducers of type I IFNs and other cytokines [29]. The data we present here indicate that in response to chronic exposure to CDT, cGAS-STING mainly regulates ISGs but not proinflammatory cytokines. At early time point of 3 days of CDT treatment, cytokine production is partially prevented in MEFs and HCECs upon STING inhibition but not cGAS. It is noteworthy that STING can be activated in a cGAS-independent manner [48], and more globally that IRF3 and NF-κB can be activated by other routes during the DDR [49]. Thus, CDT-induced proinflammatory response might rely on other DDR factors like ATM, as demonstrated in T cells treated with typhoid toxin [50]. Besides, cGAS has also been shown to play a crucial role during cellular senescence induced by DNA damage [51]. Albeit cGAS deficiency prevents CDT-mediated cell distention and senescence in HeLa, we were not able to prevent senescence through STING or cGAS inhibition in MEFs or HCECs (data not shown). As the p53 pathway, inactivated in HeLa cells, play a crucial role during senescence, perhaps cGAS-STING inhibition by itself cannot efficiently prevent CDT-induced senescence in p53 proficient cells. Alternatively, *cGAS*^*−/−*^ HeLa may be more resistant to CDT genotoxicity due to enhanced DSB repair capacity, contrary to cells treated with cGASi. Indeed, cGAS inhibits homologous recombination independently of its catalytic activity [52].

Upon chronic exposure to CDT, activation of type I IFN response by cGAS-STING may provide protective or adverse effects, depending on the physiological context. First, cGAS surveillance of MN in host cells infected with CDT-producing bacteria could elicit a rigorous immune response as previously described for microbial pathogens as DNA viruses, RNA viruses after reverse transcription, or intracellular bacteria [53]. However, CdtB from typhoid toxin rather promotes an anti-inflammatory environment in infected mice [30], which might be a consequence of proinflammatory response inhibition by type I IFN [32]. On the other hand, many ISGs, and notably those addressed in the present study, belong to the Interferon-Related DNA Damage Resistance Signature (IRDS) group, and their upregulation in cancer cells is associated with resistance to DNA damage, suppression of T cell toxicity and enhanced tumor survival [54]. Finally, modulation of the immune environment by CDT upon infection may vary with tissue or even cell type, as evidenced with cGAS downregulation in HCECs.

Persistent infection causes chronic inflammation, a driving force of tumor development [55]. CDT production during bacterial infection is thought to promote inflammation and persistent colonization, eventually contributing to tumorigenesis [9–11, 56]. However, the situation is probably more complex, as evidenced with the anti-inflammatory function of the typhoid toxin that is related to CDT by sharing the same catalytic subunit [30]. Our findings identify cGAS as a major player linking CDT genotoxic and immunomodulatory activities. cGAS is a key modulator of the innate immune response that can influence the tumor microenvironment in ways that may be detrimental or beneficial [57]. Further investigations will be necessary to unravel the role of the cGAS-dependent surveillance of MN during natural infection with CDT-producing bacteria.

### Supplementary Information

Below is the link to the electronic supplementary material.

Supplementary file1 (DOCX 24 KB)

Supplementary file3 (EPS 342 KB)

Supplementary file2 (EPS 1466 KB)

Supplementary file4 (EPS 958 KB)

Supplementary file5 (EPS 2141 KB)

Supplementary file6 (EPS 6447 KB)

Supplementary file7 (EPS 2676 KB)

Supplementary file8 (EPS 3498 KB)

## Data Availability

The microarray data from this publication have been deposited to the GEO database and assigned the identifier GSE151792 (https://www.ncbi.nlm.nih.gov/geo/query/acc.cgi?acc=GSE151792).
