# Supplementary material for: Chronic exposure to Cytolethal Distending Toxin (CDT) promotes a cGAS-dependent type I interferon response
Source: Cell Mol Life Sci. 2021 Jul 25;78(17-18):6319–35. doi: 10.1007/s00018-021-03902-x (PMC8429409; doi:10.1007/s00018-021-03902-x)
Supplement: Supplementary file 1 — Supplementary file1 (DOCX 24 KB) [file 18_2021_3902_MOESM1_ESM.docx]

**Supplemental material**

U2OS and HCT116 cells (ATCC) were cultured in Dulbecco’s Modified Eagle Medium (DMEM, Life Technologies), supplemented with 10% heat-inactivated fetal bovine serum (FBS, Gibco) and 1% antibiotics (penicillin/streptomycin). Caco-2 cells (ATCC) were cultured in DMEM supplemented with 20% heat-inactivated FBS and 1% antibiotics. The HepaRG cells (Biopredic, France) were cultured in William’s E medium (Life Technologies) supplemented with 10% FBS, 1% antibiotics, 5 μg/ml insulin, 2 mM l-glutamine and 50 μM hydrocortisone hemisuccinate. Cells were maintained at 37°C in a humidified atmosphere containing 5% CO_2_, and subcultured approximately every 2–3 days. *H. duc* CDT and *C. jej* CDT were produced and purified as previously described [56, 61].

*Supplemental Table 1: Primers for qPCR*

| Human Primers Gene | Forward (5’→3’) | Reverse (5’→3’) |
| --- | --- | --- |
| IL1β | GAACTGAAAGCTCTCCACCTCC | CCAAGGCCACAGGTATTTTGTC |
| IL6 | CCTCTTCAGAACGAATTGAC | TGCTTTCACACATGTTACTC |
| IL8 | GATTTCTGCAGCTCTGTG | GTGGAAAGGTTTGGAGTATG |
| OAS1 | CGCCTAGTCAAGCACTGGTA | CAGGAGCTCCAGGGCATA |
| MX1 | TTCAGCACCTGATGGCCTA | AAAGGGATGTGGCTGGAGAT |
| ISG15 | GCGAACTCATCTTTGCCAGTA | CCAGCATCTTCACCGTCAG |
| IFIT1 | TACCTGGACAAGGTGGAGAA | GTGAGGACATGTTGGCTAGA |
| IFIT2 | TGTGCAACCTACTGGCCTAT | TTGCCAGTCCAGAGGTGAAT |
| IFI6 | TCGCTGATGAGCTGGTCTGC | ATTACCTATGACGACGCTGC |
| IFI44 | ATGGCAGTGACAACTCGTTTG | TCCTGGTAACTCTCTTCTGCATA |
| TBP1 | TGTATCCACAGTGAATCTTGGTTG | GGTTCGTGGCTCTCTTATCCTC |
| Mouse Primers Gene | Forward (5’→3’) | Reverse (5’→3’) |
| Il6 | AGCCAGAGTCCTTCAGAGAGATACA | TTGGTCCTTAGCCACTCCTTCT |
| MX1 | GACCATAGGGGTCTTGACCAA | AGACTTGCTCTTTCTGAAAAGCC |
| ISG15 | GGTGTCCGTGACTAACTCCAT | TGGAAAGGGTAAGACCGTCCT |
| IFIT1 | CCAAGTGTTCCAATGCTCCT | GGATGGAATTGCCTGCTAGA |
| IFIT2 | AGTACAACGAGTAAGGAGTCACT | AGGCCAGTATGTTGCACATGG |
| IFI44 | AACTGACTGCTCGCAATAATGT | GTAACACAGCAATGCCTCTTGT |
| TBP1 | ACTTCGTGCAAGAAATGCTGAA | GCAGTTGTCCGTGGCTCTCT |

**Supplementary Figure legends**

**Supplementary Figure 1**. HeLa cells were exposed to CDT and subjected to colony formation assay. Results present the mean ± SD of at least 3 independent experiments.

**Supplementary Figure 2**. CDT from *Haemophilus ducreyi* (*H. duc* CDT) induces a similar type I IFN response. **(a**) *cGAS^+/+^* and *cGAS^-/-^* HeLa cells were exposed for 24 h to *H. duc* CDT 0.75 ng/ml and analyzed by immunofluorescence microscopy to quantify the frequency of micronucleated cells. (**b**) *cGAS^+/+^* and *cGAS^-/-^* HeLa cells were non-treated (NT) or exposed to *H. duc* CDT 0.75 ng/ml for 40 days and the mRNA level of the indicated genes were analyzed by RT-qPCR. (**c**) IL-6 concentration was determined by ELISA in the culture supernatant of *cGAS^+/+^* and *cGAS^-/-^* HeLa cells non-treated (NT) or exposed to *H. duc* CDT 0.75 ng/ml for 40 days. (**d**) *cGAS^+/+^* and *cGAS^-/-^* HeLa cells were non-treated (NT) or exposed to *H. duc* CDT 0.75 ng/ml for 40 days and the mRNA level of the indicated genes were analyzed by RT-qPCR. (**e**) *cGAS^+/+^* and *cGAS^-/-^* HeLa cells were exposed to HdCDT 0.75 ng/ml for 40 days and soluble fractions were analyzed by Western blotting. (**a**-**d**) Data represent the mean ± SEM of at least 3 independent experiments. Statistics were calculated by two-way ANOVA followed by Sidak’s multiple comparison test.

**Supplementary Figure 3**. HeLa cells were exposed for 24 or 72 h to etop 200 nM, campto 20 nM or MMC 100 nM and subjected to cell cycle analyzes by flow-cytometry. Data represent the mean ± SEM of 3 independent experiments. Statistics (only G2/M) were calculated by two-way ANOVA followed by Sidak’s multiple comparison test.

**Supplementary Figure 4**. HeLa cells were exposed for 24 h to CDT 0.5 ng/ml, etop 200 nM, campto 20 nM or MMC 100 nM with or without ATMi or ATRi and subjected to cell cycle analyzes by flow-cytometry. Data represent the mean ± SEM of at least 3 independent experiments.

**Supplementary Figure 5**. HeLa cells were exposed to etop 200 nM, campto 20 nM or MMC 100 nM for 24 h or 8 h, and analyzed by immunofluorescence microscopy with antibodies directed against γH2AX and pH3. Representative images (a) and quantification (b) are shown. Scale bar = 20 μm. Data represent the mean ± SEM of at least 3 independent experiments. Statistics were calculated by two-way ANOVA followed by Sidak’s multiple comparison test.

**Supplementary Figure 6**. Immunofluorescence microscopy analyses with γH2AX antibody and DAPI staining. (**a**) HeLa cells were exposed to Hduc CDT or Cj CDT for 24 h. Scale bar = 20 μm. (**b**) Indicated cell lines were exposed to Ecol CDT for 24 h. Scale bar = 10 μm.

**Supplementary Figure 7.** Mitotic defects induced by CDT in HCECs cells. (**a**, **b**) HCECs cells were exposed for 8 h to CDT and analyzed by immunofluorescence microscopy with antibodies directed against γH2AX and pH3. Representative images (a) and quantification of γH2AX positive cells (b) are shown. Data represent the mean ± SEM of at least 3 independent experiments. Statistics were calculated by two-way ANOVA followed by Sidak’s multiple comparison test. (**c**) HCECs cells were exposed for 24 h to CDT and analyzed by immunofluorescence microscopy with an antibody directed against pH3. The proportion of prometaphase, metaphase and anaphase was quantified. Data represent the mean ± SEM of at least 3 independent experiments. Statistics (only anaphase) were calculated by one-way ANOVA followed by Dunnett’s multiple comparison test.
